# Minimally invasive versus open distal pancreatectomy (LEOPARD): study protocol for a randomized controlled trial

**DOI:** 10.1186/s13063-017-1892-9

**Published:** 2017-04-08

**Authors:** Thijs de Rooij, Jony van Hilst, Jantien A. Vogel, Hjalmar C. van Santvoort, Marieke T. de Boer, Djamila Boerma, Peter B. van den Boezem, Bert A. Bonsing, Koop Bosscha, Peter-Paul Coene, Freek Daams, Ronald M. van Dam, Marcel G. Dijkgraaf, Casper H. van Eijck, Sebastiaan Festen, Michael F. Gerhards, Bas Groot Koerkamp, Jeroen Hagendoorn, Erwin van der Harst, Ignace H. de Hingh, Cees H. Dejong, Geert Kazemier, Joost Klaase, Ruben H. de Kleine, Cornelis J. van Laarhoven, Daan J. Lips, Misha D. Luyer, I. Quintus Molenaar, Vincent B. Nieuwenhuijs, Gijs A. Patijn, Daphne Roos, Joris J. Scheepers, George P. van der Schelling, Pascal Steenvoorde, Rutger-Jan Swijnenburg, Jan H. Wijsman, Moh’d Abu Hilal, Olivier R. Busch, Marc G. Besselink, for the Dutch Pancreatic Cancer Group

**Affiliations:** 1 Department of Surgery, Academic Medical Center, PO Box 22660, Amsterdam, AZ 1105 The Netherlands; 2 Department of Surgery, St Antonius Hospital, PO Box 2500, Nieuwegein, EM 3430 The Netherlands; 3 Department of Surgery, University Medical Center Groningen, PO Box 30 001, Groningen, RB 9700 The Netherlands; 4 Department of Surgery, Radboud University Nijmegen Medical Center, PO Box 9101, Nijmegen, HB 6500 The Netherlands; 5 Department of Surgery, Leiden University Medical Center, PO Box 9600, Leiden, ZA 2333 The Netherlands; 6 Department of Surgery, Jeroen Bosch Hospital, PO Box 90153, Den Bosch, ME 5200 The Netherlands; 7 Department of Surgery, Maasstad Hospital, PO Box 9100, Rotterdam, AC 3007 The Netherlands; 8 Department of Surgery, VU University Medical Center, PO Box 7057, Amsterdam, HV 1081 The Netherlands; 9 Department of Surgery, Maastricht University Medical Center, PO Box 5800, Maastricht, AZ 6202 The Netherlands; 10 Clinical Research Unit, Academic Medical Center, PO Box 22660, Amsterdam, DD 1100 The Netherlands; 11 Department of Surgery, Erasmus University Medical Center, PO Box 2040, Rotterdam, CA 3000 The Netherlands; 12 Department of Surgery, Onze Lieve Vrouwe Gasthuis, PO Box 95500, Amsterdam, HM 1090 The Netherlands; 13 Department of Surgery, University Medical Center Utrecht, PO Box 85 500, Utrecht, GA 3508 The Netherlands; 14 Department of Surgery, Catharina Hospital, PO Box 1350, Eindhoven, ZA 5602 The Netherlands; 15 NUTRIM School for Nutrition and Translational Research in Metabolism, PO Box 5800, Maastricht, AZ 6202 The Netherlands; 16 Department of Surgery, Medisch Spectrum Twente, PO Box 50 000, Enschede, KA 7500 The Netherlands; 17 Department of Surgery, Isala Clinics, PO Box 10 400, Zwolle, AB 8025 The Netherlands; 18 Department of Surgery, Reinier de Graag Gasthuis, PO Box 5011, Delft, GA 2600 The Netherlands; 19 Department of Surgery, Amphia Hospital, PO Box 90 158, Breda, RK 4800 The Netherlands; 20 Department of Surgery, Southampton University Hospital NHS Foundation Trust, Southampton, SO166YD UK; 21 Department of Surgery, Academic Medical Center, PO Box 22660, Amsterdam, DD 1100 The Netherlands

**Keywords:** Minimally invasive, Laparoscopic, Robot-assisted, Distal pancreatectomy, Pancreatic surgery, Pancreatic cancer

## Abstract

**Background:**

Observational cohort studies have suggested that minimally invasive distal pancreatectomy (MIDP) is associated with better short-term outcomes compared with open distal pancreatectomy (ODP), such as less intraoperative blood loss, lower morbidity, shorter length of hospital stay, and reduced total costs. Confounding by indication has probably influenced these findings, given that case-matched studies failed to confirm the superiority of MIDP. This accentuates the need for multicenter randomized controlled trials, which are currently lacking. We hypothesize that time to functional recovery is shorter after MIDP compared with ODP even in an enhanced recovery setting.

**Methods:**

LEOPARD is a randomized controlled, parallel-group, patient-blinded, multicenter, superiority trial in all 17 centers of the Dutch Pancreatic Cancer Group. A total of 102 patients with symptomatic benign, premalignant or malignant disease will be randomly allocated to undergo MIDP or ODP in an enhanced recovery setting. The primary outcome is time (days) to functional recovery, defined as all of the following: independently mobile at the preoperative level, sufficient pain control with oral medication alone, ability to maintain sufficient (i.e. >50%) daily required caloric intake, no intravenous fluid administration and no signs of infection. Secondary outcomes are operative and postoperative outcomes, including clinically relevant complications, mortality, quality of life and costs.

**Discussion:**

The LEOPARD trial is designed to investigate whether MIDP reduces the time to functional recovery compared with ODP in an enhanced recovery setting.

**Trial registration:**

Dutch Trial Register, NTR5188. Registered on 9 April 2015

**Electronic supplementary material:**

The online version of this article (doi:10.1186/s13063-017-1892-9) contains supplementary material, which is available to authorized users.

## Background

Minimally invasive surgery, which includes both laparoscopic and robot-assisted surgery, has undoubtedly been one of the most significant advances in surgery during the past decade and is now the preferred approach for many surgical procedures [1–8]. Minimally invasive surgery aims to reduce postoperative pain and complications and shorten the time to functional recovery [1–8].

Minimally invasive distal pancreatectomy (MIDP) was first described in 1994 by Cuschieri et al. [9] Since then, several observational cohort studies from expert centers have suggested that MIDP is safe, feasible and cost-effective in the treatment of benign, premalignant and malignant lesions of the distal pancreas [10, 11]. These suggestions were confirmed by several systematic reviews of cohort studies that reported reductions in intraoperative blood loss, blood transfusion, complications, wound infections and shorter hospital stay, and an increased rate of spleen preservation, all in favor of the minimally invasive approach [10, 11].

Regarding oncologic parameters, including resection margins and lymph node retrieval, the minimally invasive approach is considered to be at least non-inferior to conventional open distal pancreatectomy (ODP) [12–15]. Case-matched studies, however, have not confirmed the presumed benefits of MIDP [10], indicating that confounding by indication has clearly played a role in the reported results in cohort studies. This is confirmed by a difference in baseline characteristics as reported in many series, such as smaller tumor size in the MIDP group [11]. It is therefore currently unknown whether MIDP, as a routine strategy, actually offers clinically relevant advantages over ODP. Most cohort series were performed in high-volume expert centers, so it is uncertain whether these outcomes of MIDP are generalizable [10]. Hence, randomized controlled trials are needed in order to provide high-quality evidence on the benefits of MIDP over ODP.

Minimally invasive approaches are often assumed to be more expensive than open surgery, because of the need for expensive surgical equipment and sometimes prolonged operative times. However, for several procedures, such as appendectomy, cholecystectomy and colectomy, no difference in overall costs has been demonstrated, mainly because reduced hospital stay balanced the higher procedural costs [16]. The current literature is unclear as to the cost difference between MIDP and ODP [10].

In 2014, the Dutch Pancreatic Cancer Group (DPCG) initiated the longitudinal assessment and realization of minimally invasive pancreatic surgery in The Netherlands (LAELAPS) training program to implement minimally invasive pancreatic surgery in The Netherlands [17]. This program follows the preferred steps of surgical innovation according to the innovation, development, exploration, assessment and long-term study (IDEAL) statement [18–20].

First, a nationwide retrospective analysis on distal pancreatectomy was performed in order to assess the utilization and outcomes of MIDP and ODP [21]. In this retrospective multicenter study in 633 patients, MIDP appeared to be at least non-inferior to ODP, but the nationwide underuse of MIDP (10%) and high conversion rate (38%) revealed clear room for improvement. The subsequent nationwide LAELAPS training program included detailed technique standardization and description, video training and proctoring [17]. In the period after training, there was a sevenfold increase in the use of MIDP, and blood loss and conversion rates decreased even during surgery on more complex tumors, including more pancreatic ductal adenocarcinomas. The 8% conversion rate, 17% Clavien-Dindo grade III or higher complication rate, 6 days hospital stay and 0% 30-day mortality in the period after training were comparable to results from expert centers in the UK and USA [11]. According to the IDEAL framework, a randomized controlled trial should be the next step [18–20]. Accordingly, the aim of the LEOPARD trial is to compare the outcomes, including time to functional recovery, complications, quality of life and costs, after MIDP with ODP within an enhanced recovery setting.

## Methods

### Design

The LEOPARD trial is a randomized controlled, parallel-group, patient-blinded, multicenter, superiority trial investigating the effectiveness of MIDP versus ODP for treatment of symptomatic benign, premalignant or malignant disease of the distal pancreas in an enhanced recovery setting. Eligible patients will be randomized equally to either MIDP or ODP. Splenectomy will be performed only when indicated for oncological or technical reasons.

### Trial population

All adult patients with an indication for elective distal pancreatectomy (with or without splenectomy) because of suspected or proven symptomatic benign, premalignant or malignant disease of the distal pancreas, from 17 centers performing pancreatic surgery in The Netherlands will be assessed for eligibility.

### Inclusion criteria

The inclusion criteria are as follows:

- Age equal to or above 18 years
- Indication for elective distal pancreatectomy (with or without splenectomy) because of proven or suspected left-sided symptomatic benign, premalignant or malignant disease
- Tumor meeting the Yonsei criteria [22] (tumor or cyst confined to the pancreas with an intact posterior pancreatic fascial layer and the tumor located at least 1 cm from the celiac axis)
- Sufficiently fit to undergo distal pancreatectomy according to the surgeon and anesthetist

### Exclusion criteria

The exclusion criteria are as follows:

- Tumor or cyst larger than 8 cm
- Surgical intervention (resection or ablation) of other organs besides the distal pancreas or spleen are required (cholecystectomy is allowed)
- Previous radiotherapy as treatment of pancreatic cancer
- Chronic pancreatitis (according to the M-ANNHEIM criteria)
- Pregnancy
- Participation in another study with interference in study outcomes

### Randomization

**Fig. 1:**
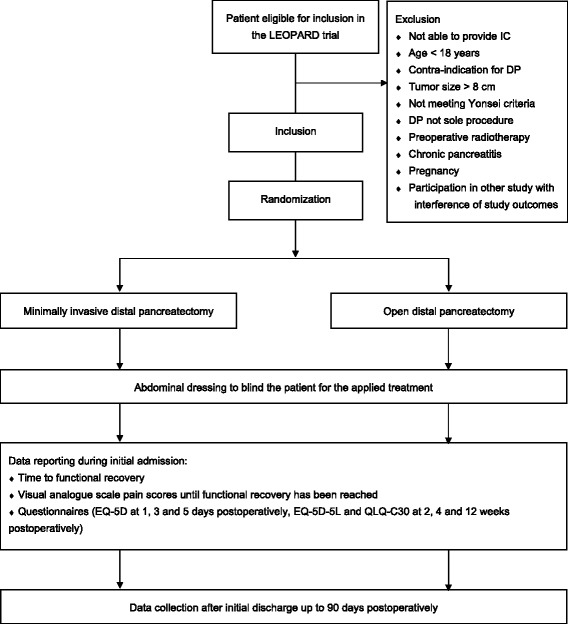
Minimally invasive versus open distal pancreatectomy for symptomatic benign, premalignant and malignant disease (*LEOPARD*) trial flow diagram according to standard protocol items: recommendations for interventional trials (SPIRIT) [45]. *IC* informed consent, *DP* distal pancreatectomy, *EQ-5D-5 L* Euro-QoL five health dimensions questionnaire, *QLQ-C30* quality of life questionnaire including 30 questions

Patients will be randomized centrally by the trial coordinators using an online randomization module (ALEA, Clinical Research Unit, Academic Medical Center, Amsterdam, The Netherlands) in a 1:1 ratio between MIDP and ODP, as shown in Fig. 1. Randomization will be stratified by center to balance differences in the surgical procedure and general treatment regimens and by the indication for surgery (malignant versus non-malignant disease). Permuted-block randomization will be used to provide treatment allocation in equal proportions, with block sizes that will be subject to random variation. This will be concealed to all investigators involved in the trial.

### Intervention: minimally invasive distal pancreatectomy

Patients are under general anesthesia for minimally invasive distal pancreatectomy, and epidural anesthetic is not mandatory. The patient is placed in a supine position, left side 30 degrees elevated with legs apart. In total, four to five (2 × 12 mm and 2 × 5 mm to 3 × 5 mm) trocars are placed in a semicircular fashion, centered around the supra-umbilical camera. The surgeon stands at the patient’s right side, the assistant controlling the camera stands between the patient’s legs and the assistant surgeon stands at the patient’s left side.

The omental bursa is opened by dividing the gastrocolic ligament using an ultrasonic device (Harmonic ACE® + 7 Shears, Ethicon Inc., Cincinnati, OH, USA), or standard of care. Division of the short gastric arteries depends on the indication of spleen preservation. In patients with spleen-preserving surgery, the left gastro-epiploic artery and short gastric vessels are fully preserved. The posterior fundus of the stomach is retracted. The lesion is identified, either visually or with laparoscopic ultrasound. The splenic artery is identified at the superior margin of the pancreas and slung with a vessel loop which is secured with a Hem-o-lok® clip (Teleflex Medical, Weck Drive, Research Triangle Park, NC, USA). Optionally, in spleen-preserving surgery, a laparoscopic bulldog clamp can be placed on the artery to reduce blood loss during the procedure. Transection is postponed until the anatomy (especially the hepatic artery and celiac trunk) is confirmed. Alternatively, the stomach is retracted caudally and the common hepatic artery, celiac trunk and splenic artery are identified cranial to the stomach.

The caudal margin of the pancreas is mobilized and the inferior and superior mesenteric veins are identified. An umbilical tape is placed under the pancreas between the lesion and the spleen and secured with a Hem-o-lok® clip. In the case of suspected malignancy this umbilical tape includes Gerota’s fascia as described previously [23]. The same procedure is performed at the right side of the tumor, potentially over the portomesenteric vein. In the case of malignancy, lymphadenectomy is performed according to the International Study Group on Pancreatic Surgery (ISGPS) consensus [24]. If the tumor is localized in the pancreatic body, lymphadenectomy also involves station 9 (around the celiac trunk).

Dissection according to the radical antegrade modular pancreatosplenectomy (RAMPS) method, as described by Strasberg [25], which includes lymph node dissection from the common hepatic artery to the celiac trunk and splenic artery, is advised in the case of malignancy. The left gastric artery is preserved if possible. In the case of benign and premalignant lesions attempts are made to preserve the spleen. These vessels are either completely spared (Kimura’s technique [26]) or if complete preservation of the splenic vessels is not feasible they will be divided using either Hem-o-lok® clips (at least two clips on the patient’s side) or stapling devices, so the spleen only receives blood via the short gastric arteries (Warshaw’s technique [27]). Splenectomy is performed in the case of gross splenic ischemia. Splenectomy is routinely performed in the case of malignant lesions or risk of malignancy.

The pancreas is divided using an endostapler with the staple cartridge size adapted to the thickness of the pancreas. A graded progressive compression technique is used as described by Asbun et al. [28]. Additional suturing of the pancreatic stump, covering the stump with tissue or a patch, or staple-line reinforcement is allowed. A medial-to-lateral approach is used during the mobilization of the pancreas. The pancreatic tail (with or without the spleen) is put into a leak-proof retrieval bag and extracted using a Pfannenstiel incision or an enlarged trocar incision. A surgical drain is placed near the pancreatic remnant. In the case of splenectomy this drain is placed via a long loop through the left upper quadrant with additional side holes to drain the splenic bed. The same approach can be followed with the DaVinci® console. Small variations according to the surgeon’s preference are allowed, but have to be recorded in the case report form.

### Control: open distal pancreatectomy

Patients undergo multimodal pain therapy with either an epidural catheter or wound catheters with patient-controlled analgesia [29]. Subcostal or midline laparotomy is performed. The steps taken are essentially similar to minimally invasive surgery but variation in technique is expected to be larger. In the case of benign or premalignant disease an attempt at splenic preservation is made, preferably using Kimura’s technique [26] (preservation of splenic vessels), but otherwise using Warshaw’s technique [27] (transecting splenic vessels). Tissue dissection during ODP, except for pancreatic transection, will be performed using a standardized ultrasonic device (Harmonic ACE® + 7 Shears, Ethicon Inc., Cincinnati, OH, USA, or similar device) within the LEOPARD trial. The pancreas is transected with a stapler. Alternatively, transection with ultrasonic devices, diathermia or sharp transection with suturing is allowed, as it is not expected that this will influence perioperative outcomes [30]. Additional suturing of the pancreatic stump, covering the stump with tissue or a patch, or staple-line reinforcement is allowed. Small variations according to the surgeon’s preference are allowed, but have to be recorded in the case report form.

### Conversion from MIDP to ODP

Conversion is defined as any MIDP (laparoscopic or robot-assisted) in which an incision is used for reasons other than trocar placement or specimen extraction. Patients allocated to MIDP who undergo intraoperative conversion to ODP will still be analyzed in the MIDP group according to intention-to-treat principles. Reasons for conversion will be registered.

### Patient blinding

**Fig. 2:**
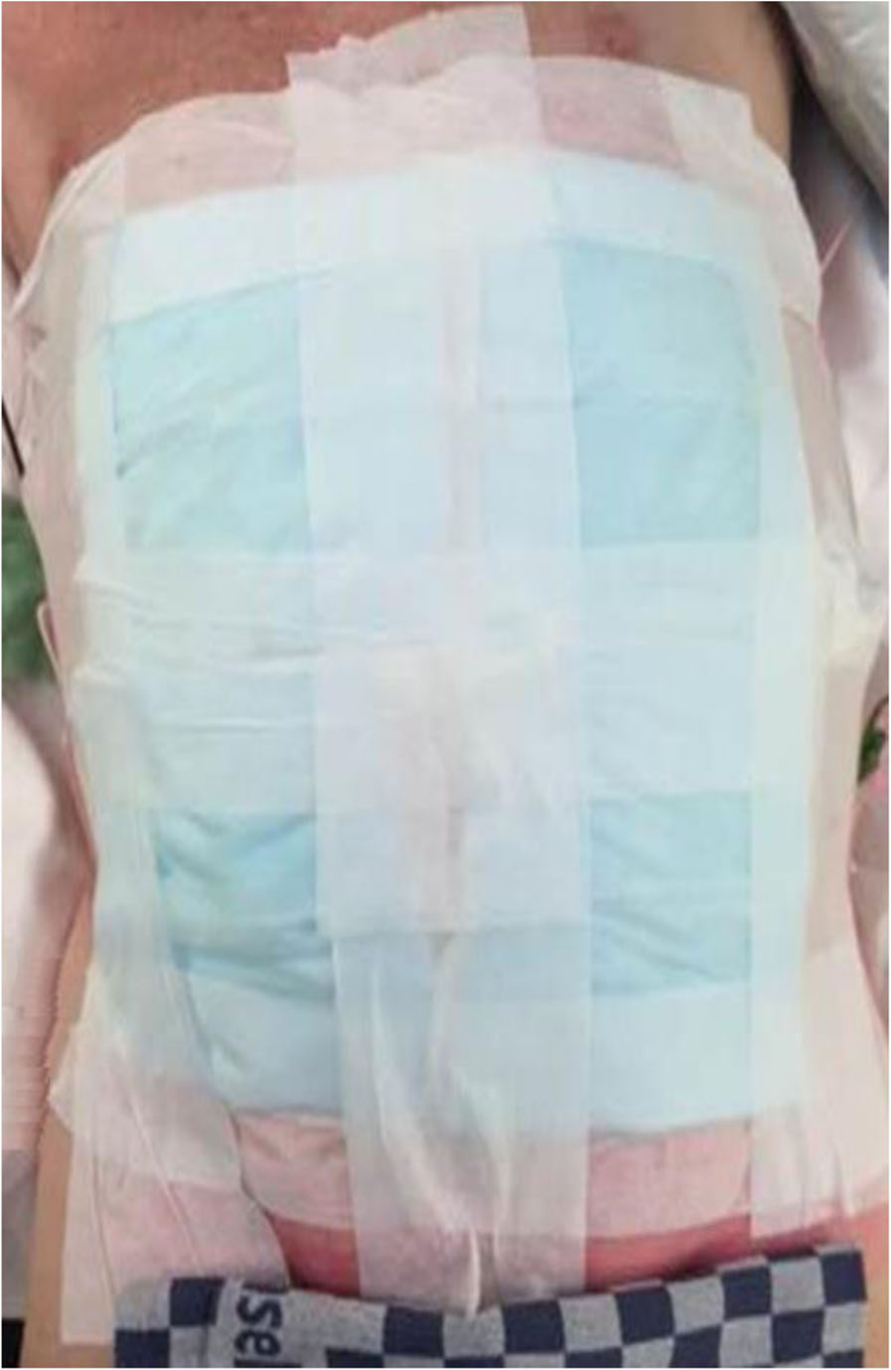
Abdominal dressing used to blind the patient for the type of distal pancreatectomy

Patients will be blinded using a large (30 × 30 cm) abdominal dressing, which is administered in the operating room immediately after the operation (Fig. 2). Patients are blinded until functional recovery has been reached or at least up to day 5 postoperatively. Earlier removal of the dressing is allowed for urgent medical reasons. Previous trials in The Netherlands have found this approach feasible [8, 31]. Full blinding of medical and nursing ward staff would include masking surgical notes to the hospital electronic patient system and is not considered feasible in all 17 centers.

### General treatment regimen

Postoperative care is similar in both arms and based on enhanced recovery after surgery principles, which include early mobilization and expanding oral intake as desired by the patient. When patients are functionally recovered, they are essentially medically ready to be discharged. The final date of discharge will be determined by the local treating team.

### Primary outcome

The primary outcome is time to functional recovery (days). Functional recovery will be daily assessed by the nurses, ward physicians and the trial coordinators and is reached when all of the following criteria are met:

- Adequate pain control with oral analgesia only
- Restoration of mobility to an independent level (or to preoperative level if previously impaired)
- Ability to maintain sufficient caloric intake (minimum of 50% required calories); absence of intravenous fluid administration
- No signs of active abdominal infection (in the case of abdominal infection this item is met when serum C-reactive protein is below 150 mg/L and the patient has no fever)

### Secondary outcomes

The secondary outcomes of this trial include operative outcomes (type of approach, vascular tumor involvement, tumor involvement in multiple organs, type of primary and secondary surgeon (resident, fellow or surgical specialist), conversion and reason for conversion, method of pancreatic transection, spleen preservation (including technique: Kimura/Warshaw), vessel resection, intraoperative blood transfusion, administration of somatostatin analogs, intra-operative complications, operative time (from first incision to full skin closure), total duration of the procedure and intraoperative blood loss, type of analgesia), postoperative outcomes (pain management, complications (e.g. postoperative pancreatic fistula, post-pancreatectomy hemorrhage, delayed gastric emptying and surgical site infection), postoperative intervention (radiologic, endoscopic, surgical), postoperative blood transfusion, intensive care unit admission, length of hospital stay, readmission, time to start adjuvant chemotherapy (in the case of malignant disease), mortality and Clavien-Dindo scores of all individual complications), pathology parameters (resected specimen length, tumor size, histopathological diagnosis, resection margins (including transection, anterior circumferential and posterior circumferential margins, in the case of malignancy), lymph node retrieval, tumor-positive lymph node retrieval (in the case of malignancy), neural and vascular tumor invasion), costs (intra-operative and postoperative costs) and quality of life.

### Data collection and patient follow up

Baseline data (on age, sex, performance status (Karnofsky score), American Society of Anesthesiologists physical status, body mass index, malnutrition universal screening tool score (MUST), ERAS mobility scale [31, 32], diabetes mellitus, previous abdominal surgery, preoperative imaging including tumor size and involvement of other organs, preoperative diagnosis, indication for hospitalization, indication for surgery) will be collected before randomization using standardized case report forms. Data on the primary and secondary outcomes will be collected from randomization up to 90 days postoperatively, by the local treating physicians or the trial coordinators using standardized case report forms. The case report forms and the database will be crosschecked with source data by the trial coordinators.

Patients will be asked to complete validated questionnaires (EQ-5D-5 L and QLQ-C30) at 2 weeks, 4 weeks and 12 weeks postoperatively (Fig. 1). One year after surgery, patients will be asked whether they are totally recovered and will be asked to complete questionnaires on quality of life (Euro-QoL 5 health dimensions questionnaire (EQ-5D-5 L) and Quality of life questionnaire including 30 questions (QLQ-C30)), complications and whether they would recommend MIDP or ODP to a friend or family member. Long-term follow-up results of the LEOPARD trial (including complications and quality of life) will be published separately.

### Definitions

The distal pancreas is defined as the proportion of the pancreas located at the left of the portomesenteric vein. Complications are classified using the Clavien-Dindo score [33]. Major complications are defined as a Clavien-Dindo score III or higher. Postoperative pancreatic fistula [34], delayed gastric emptying [35] and post-pancreatectomy hemorrhage [36] are classified using the International Study Group on Pancreatic Fistula (ISGPF) and the International Study Group on Pancreatic Surgery (ISGPS) definitions, respectively. Surgical site infection is classified according to the Centers for Disease Control and Prevention definition [37]. In the case of malignant pancreatic disease, resection margins, including transection and circumferential margins, are classified by a margin to tumor distance ≥1 mm (R0), a margin to tumor distance <1 mm (R1) or a macroscopically positive margin (R2) [38]. Tumor, node, metastases (TNM) status is classified according to the American Joint Committee on Cancer (AJCC) classification (7^th^ edition).

### Quality and safety

Surgeons will only be allowed to participate in the LEOPARD trial when they have completed LAELAPS training in MIDP [17] and have performed >50 advanced minimally invasive advanced gastrointestinal procedures, >20 distal pancreatectomies (either MIDP or ODP) and >5 MIDPs. An advanced minimally invasive gastrointestinal procedure is defined as any minimally invasive gastrointestinal procedure beyond diagnostic laparoscopy, cholecystectomy and appendectomy. If surgeons do not meet all of the predefined criteria, they will be assisted by an experienced MIDP surgeon (defined as having performed >20 MIDPs) as often as necessary until the criteria are met. Each participating surgeon will record a video of the first MIDP performed in the trial. This video will be shortened to enable efficient scoring of the operator’s skills and scored by an expert MIDP surgeon, who will score the video (blinded for the surgeon and clinical outcomes) using the method described by Birkmeyer et al. [39]. The expert surgeon will assess each video in five domains of technical skills (gentleness, tissue exposure, instrument handling, time and motion and flow of the operation). Each domain will be rated on a scale of 1–5, where 1 indicates the skill expected of a general surgical resident and 5 the skill of a master surgeon in the procedure of minimally invasive distal pancreatectomy.

All adverse events reported spontaneously by the patient or observed by the investigator or his staff will be recorded up to 90 days postoperatively. Serious adverse events occurring within 90 days after treatment will be recorded. Only serious adverse events from a predefined list will be reported through a Web portal to the central committee on research involving human subjects (in Dutch: central *Commissie Mensgebonden Onderzoek*) and the accredited institutional review board (www.toetsingonline.nl). This includes serious adverse events that necessitate intensive care unit admission, necessitate surgical intervention, necessitate readmission or result in mortality (for any reason). The remaining events are recorded in a yearly overview list. An independent data safety monitoring board will meet to investigate the safety parameters after every 25 included patients. This data safety monitoring board comprises one independent statistician/epidemiologist (Chair), one independent gastroenterologist and one independent surgeon. The result(s) of the data safety monitoring board meeting will be sent to all participating physicians involved in this trial. Furthermore, the result of the meeting will be relayed to the trial steering committee. The advice of the data safety monitoring board will only be sent to the sponsor of the trial.

### Statistical aspects

#### Sample size calculation

The LEOPARD trial is designed as a superiority trial, hypothesizing that the time to functional recovery is significantly shorter after MIDP compared with ODP. Based on the most recently published meta-analysis [11] and Dutch nationwide data, a time to functional recovery of 8 days in the control group (ODP) versus 6 days in the intervention group (MIDP) is expected with a standard deviation of 3 days. The significance level (α) is set at 0.05 and power (1-β) at 80%. The sample size needed in each arm, calculated using the independent samples *t* test, is 36 patients. Including 15% crossover from the MIDP to ODP and a rate of 2% loss to follow up (based on previous studies), a total of 51 patients will be randomized in each group, so a total of 102 patients will be randomized in the LEOPARD trial.

After completion of data collection in the first 75 patients, the data safety monitoring board will assess potential data skewness and homogeneity of variance for time to functional recovery. When a non-parametric test seems indicated for the comparison of MIDP versus ODP on time to functional recovery, the total sample size will become the calculated sample size (102) divided by the asymptomatic relative efficiency parameter of the Mann–Whitney *U* test (0.955). In that case, a total of 108 patients will be randomized in the LEOPARD trial. Procedures performed using the robot DaVinci® Surgical System are allowed in this trial, but will be analyzed separately during the cost analysis.

#### Statistical analysis

Primary and secondary outcomes will be crosschecked with data from primary sources and a blinded adjudication committee will check them against the definitions, which were established before the start of this trial. Categorical variables will be compared using the chi-square or Fisher’s exact test as appropriate, and values will be expressed as proportions. The distribution of continuous variables will be determined using visual inspection and the Kolmogorov-Smirnov test.

For comparison of normally distributed continuous variables the independent samples *t* test will be used and values will be expressed as means with standard deviations. Continuous non-normally distributed variables will be compared using the Mann–Whitney *U* test and values will be expressed as medians with interquartile ranges. Measures of association will be expressed as relative risks with 95% confidence intervals. A difference with a two-tailed *P*-value <0.05 will be considered statistically significant.

Analyses will be performed based on intention-to-treat principles, meaning that converted MIDP will be assessed in the MIDP group. A multivariable linear regression model will be used to assess potential differences between groups in the primary outcome in the presence of potentially confounding factors. Linear mixed modeling will be applied to estimate differences between groups in successive EQ-5D-5 L and QLQ-C30 assessments over time. For exploratory purposes a secondary analysis will be performed comparing outcomes in patients with malignant versus non-malignant disease, comparing completed MIDP (i.e. no conversion) versus ODP and comparing time to functional recovery between MIDP and ODP in patients with complications (Clavien-Dindo score ≥ III) and without complications.

### Dissemination policy

The results of the LEOPARD trial will be submitted to a peer-reviewed journal regardless of the outcome. Authorship will be based on international guidelines. Participants who do not fulfil the authorship criteria will be listed in PubMed as ‘collaborators’.

## Discussion

The LEOPARD trial is a multicenter randomized controlled trial designed to assess whether MIDP reduces the time to functional recovery compared with ODP, in an enhanced recovery setting. LEOPARD was initiated by the Dutch Pancreatic Cancer Group, a national collaboration of surgeons, gastroenterologists, medical oncologists, pathologists, (interventional) radiologists, dietitians and nurses, which aims to improve the treatment of benign, premalignant and malignant pancreatic disease.

The LEOPARD trial is the first multicenter randomized controlled trial comparing MIDP to ODP. On the World Health Organization trial registry website, incorporating all (inter)national trial registries, there are only two single-center randomized trials reported in this field. The first trial (LAPOP) with a total sample size of 60 patients is from Sweden and is planned to be completed in 2020 [40]. Patients are not blinded to the intervention in this trial. The second trial is from the USA and was never started [41]. Randomization is essential to exclude the strong influence of selection bias. The LEOPARD trial is expected to be the first multicenter randomized trial to report high-level evidence, which will be of significant value for clinical practice and guideline development. Furthermore, this project will not only corroborate the demonstrated advances in reducing hospital stay and morbidity, but will also generate evidence on quality of life and costs. Several recently published studies have suggested that the generalizability of the benefits of MIDP remains undefined and randomized controlled multicenter trials are therefore needed [10–12].

There is no obvious clinical diversity in patients diagnosed with left-sided pancreatic disease, as these lesions are found in both women and men, at all ages, with different ethnicity. All of these demographic diversities, except for children, will be fully represented in the LEOPARD trial and the investigated procedures are uniform for all patients. The LEOPARD trial hypothesizes a superior value of MIDP for time to functional recovery and therefore all indications for elective distal pancreatectomy (with or without splenectomy) can be included. The Yonsei criteria will ensure oncological safety in patients diagnosed with pancreatic cancer within this trial, as it increases the probability of achieving R0 tumor resection margins [22]. Additional analyses will be performed to investigate the outcomes in several subgroups of patients (e.g. those with non-malignant versus malignant disease and those with and without complications).

Within the LEOPARD trial, laparoscopic surgery and robot-assisted surgery are both considered minimally invasive and therefore both are allowed in the “minimally invasive” arm. Furthermore, no clear differences in hospital stay and morbidity have been shown for one versus the other [10].

Patient blinding should be performed (if possible) in randomized controlled trials to decrease the influence of several types of bias and should especially be considered in trials with patient-reported outcome measures as the primary and/or secondary outcome [42]. Functional recovery is partly patient-reported (e.g. pain perception) and is easily influenced by the patient’s expectation. Patient blinding is therefore essential to eliminate the Hawthorne effect, which means that if patients know that they have been allocated to a specific arm, their behavior and response will consequently be influenced [43]. Patient blinding in randomized controlled trials in abdominal surgery has been successfully performed in previous trials [8, 31], but its impact remains to be assessed [44].

In conclusion, the LEOPARD trial is a multicenter randomized controlled trial investigating time to functional recovery after MIDP versus ODP. This trial aims to provide level-1 evidence on the added value of the minimally invasive approach. When this hypothesis is confirmed, it will enhance the worldwide implementation of MIDP and consequently improve patient outcomes.

## Trial status

The first patient was randomized on 9 April 2015. At the time of protocol submission (9 November 2016), all centers were actively recruiting patients for the trial and 78 of 102 patients (76%) have been randomized, which means that recruitment is on schedule.

## Additional file

Additional file 1: SPIRIT 2013 checklist: recommended items to address in a clinical trial protocol and related documents. (DOC 112 kb)

## Abbreviations

AJCC: American Joint Committee on Cancer
DPCG: Dutch Pancreatic Cancer Group
EQ-5D-5L: Euro-QoL five health dimensions questionnaire
ERAS: Enhanced recovery after surgery
IDEAL: Idea, development, exploration, assessment and long-term study
ISGPF: International Study Group on Pancreatic Fistula
ISGPS: International Study Group on Pancreatic Surgery
LAELAPS: Longitudinal assessment and realization of minimally invasive pancreatic surgery in The Netherlands
LEOPARD: Minimally invasive versus open distal pancreatectomy for symptomatic benign, premalignant and malignant disease
MIDP: Minimally invasive distal pancreatectomy
MUST: Malnutrition universal screening tool
ODP: Open distal pancreatectomy
QLQ-C30: Quality of life questionnaire including 30 questions
RAMPS: Radical antegrade modular pancreatosplenectomy
SPIRIT: Standard protocol items: recommendations for interventional trials
USA: United States of America
WMO: Medical Research Involving Human Subjects Act
